# Socioeconomic and Clinical Determinants Driving Access to BRCA Genetic Testing in Cancer : A Case-Control Study Using Observational Electronic Health Records Across Multiple Sites

**DOI:** 10.64898/2026.05.14.26353261

**Published:** 2026-05-21

**Authors:** Qiang Yang, Chengrong Wang, Charite Ricker, Sandra G. Suther, Qianqian Song, Sarah Khan, Yi Guo, Thomas J George, Mattia Prosperi, Rui Yin

## Abstract

**Importance:**

*BRCA* genetic testing is critical for cancer risk assessment, treatment and personalization, yet substantial underutilization persists. Socioeconomic and clinical factors may strongly influence testing uptake; therefore, identifying the potential drivers to *BRCA* testing and treatment is essential for addressing gaps in access, increasing retention into care, and improving cancer outcomes.

**Objective:**

To quantify the putatively causal effects of SDoH on *BRCA* genetic testing among individuals with breast, ovarian, pancreatic, and prostate cancers and to develop a predictive model to identify patients at risk for underuse of testing.

**Design, Setting, and Participants:**

This observational case-control study used data from a large multistate clinical research data network covering southern US (2012-2023). The network contained records of more than 26 million individuals and was linked with ZIP code-level SDoH variables derived from national socioeconomic datasets. Adults diagnosed with breast, ovarian, pancreatic, or prostate cancer were eligible for cases (received BRCA testing) or controls (did not receive BRCA testing, matched by cancer diagnosis).

**Exposure:**

SDoH categories, including economic conditions, education, healthcare access, neighborhood conditions, and social connectedness.

**Main Outcomes and Measures:**

The primary outcome was receipt of *BRCA* genetic testing after cancer diagnosis.

**Results:**

Among 3,279 people diagnosed with cancer, 748 received *BRCA* testing and 2,531 served as controls. Study population’s mean [SD] age was 66.8 [15.7] years; 1,758 were women [53.6%], 2,238 [69.6%] were White and 616 [18.8%] were Black or African American. Breast (1,420 [42.8%]) and prostate (1,342 [40.9%]) cancers were the most common diagnoses, followed by pancreatic (242 [7.4%]), ovarian (238 [7.2%]), and multiple cancers (55 [1.7%]). Upon adjusting for potential confounding, higher educational attainment (odds ratio [OR], 1.19), public-sector employment (OR, 1.42), neighborhood safety (OR, 1.28), and social participation (OR, 1.72) showed an increased likelihood of undergoing *BRCA* testing, whereas economic instability, including housing cost burden and reliance on public insurance, had an effect of reduced testing. A random forest classifier demonstrated good discriminative performance (AUROC, 0.776) to predict cancer patients who were likely to take *BRCA* testing, where nativity, language, and residential stability ranked among the most influential social determinants according to SHapley Additive exPlanations (SHAP) analysis.

**Conclusions and Relevance:**

In this observational case-control study, SDoHs were strongly associated with receipt of *BRCA* genetic testing among people with cancer. These findings suggest that disparities in genetic testing may reflect structural and social barriers rather than differences in clinical eligibility alone. Efforts to improve equitable access to genetic testing may benefit from integrating social-context information into clinical workflows and targeting outreach or navigation strategies toward socially disadvantaged populations.

**Key Points:**

**Question**

Do socioeconomic and clinical circumstances contribute to care access gaps to *BRCA* genetic testing among people with breast, ovarian, prostate, and pancreatic cancers?

**Findings**

In a case-control study of 3,279 adults with cancer from a large multi-state US clinical research data network, multiple social determinants of health, including economic conditions, education, healthcare access, neighborhood conditions, and social connectedness, were identified as key determinants of *BRCA* genetic testing uptake, with variation across age and sex groups.

**Meaning**

Improving equal access to *BRCA* genetic testing may require interventions that address financial barriers and strengthen social, and community supports that influence engagement with genetic services.

## Full Text Availability

The license terms selected by the author(s) for this preprint version do not permit archiving in PMC. The full text is available from the preprint server.

